# Mature Gastric Teratoma in a 3‐Month‐Old Male Infant Presenting With Progressive Abdominal Distension: A Rare Pediatric Tumor With Complex Diagnostic and Surgical Considerations

**DOI:** 10.1002/ccr3.72298

**Published:** 2026-03-15

**Authors:** Syed Iftikhar Rahim, Mahnoor Nazir Afridi, Arjun Singh, Muhammad Waqas, Sadaf Faryal, Ang Raj Karan, Asim Shah, Suleman Khan, Aizaz Anwar Khalid, Hammad Iftikhar, Mohammed Hammad Jaber Amin

**Affiliations:** ^1^ Lady Reading Hospital Peshawar Pakistan; ^2^ Khyber Medical College Peshawar Pakistan; ^3^ Peshawar Medical College Peshawar Pakistan; ^4^ Department of Medicine Alzaiem Alazhari University Khartoum Sudan

**Keywords:** gastric teratoma, infant abdominal mass, mature teratoma, pediatric germ cell tumor, surgical excision

## Abstract

Gastric teratoma is a sporadic pediatric tumor, usually appearing in early infancy. We present a 3‐month‐old male infant with progressive abdominal distension, chronic constipation, and a firm upper‐abdominal mass. Imaging showed a large heterogeneous lesion originating from the greater curvature of the stomach, with mixed solid and cystic components suggestive of a teratoma. The mass was entirely removed via laparotomy, and histopathology confirmed a mature gastric teratoma containing tissues from all three germ layers without malignant features. The postoperative recovery was smooth, with rapid symptom improvement. This case emphasizes the importance of considering gastric teratoma in infants with unexplained abdominal distension and supports complete surgical removal as an effective management approach in this case.

## Key Clinical Message

Mature gastric teratoma is a rare cause of progressive abdominal distension in infants. Early imaging is essential for identifying a heterogeneous gastric mass, and complete surgical excision offers an excellent prognosis with rapid postoperative recovery.

## 1 Introduction

Gastric teratomas are extremely rare pediatric germ cell tumors, accounting for < 1% of all childhood teratomas. These tumors are exceptionally uncommon, with only a few cases documented in the literature. They most frequently occur during the neonatal or early infancy period, usually within the first few months of life, and are rarely seen in older children or adults [1]. Gastric teratomas are complex and typically consist of tissues from all three germ cell layers: ectoderm, mesoderm, and endoderm. These tumors can contain various differentiated tissues such as hair, teeth, bone, and even neural tissue, reflecting their origin from pluripotent cells [2]. Diagnosis is often made through imaging techniques, including ultrasonography or contrast‐enhanced computed tomography (CT). These imaging methods reveal a heterogeneous abdominal mass, which may include both solid and cystic areas as well as calcifications characteristic of teratomas. The combination of these features helps distinguish gastric teratomas from other abdominal masses [3]. Clinically, infants with gastric teratomas typically present with symptoms like progressive abdominal distension, often accompanied by a palpable abdominal mass. This mass can vary in size and is usually the initial sign prompting further diagnostic evaluation [4]. Treatment mainly involves complete surgical removal of the tumor, and the prognosis after surgery is generally very favorable, with most patients making a full recovery. However, due to the rare risk of malignant transformation, long‐term follow‐up is recommended to monitor for recurrence or signs of malignancy after surgery [5].

## 2 Case Presentation

A 3‐month‐old male infant was brought to the pediatric clinic by his parents due to persistent abdominal bloating, chronic constipation, and feeding difficulties. These symptoms have persisted for about 2 months. He was born full‐term through a normal vaginal delivery with no significant neonatal issues. His growth and development are otherwise normal, but the parents are concerned about the increasing abdominal size and irregular bowel movements.

On physical examination, the infant was alert and showed no signs of acute distress. The abdomen was distended, and a firm, nontender mass was felt in the left upper quadrant, which appeared to involve the stomach. There was no palpable organomegaly, and bowel sounds were present. The infant's vital signs were typical, with no signs of dehydration or malnutrition.

## 3 Investigations

**FIGURE 1:**
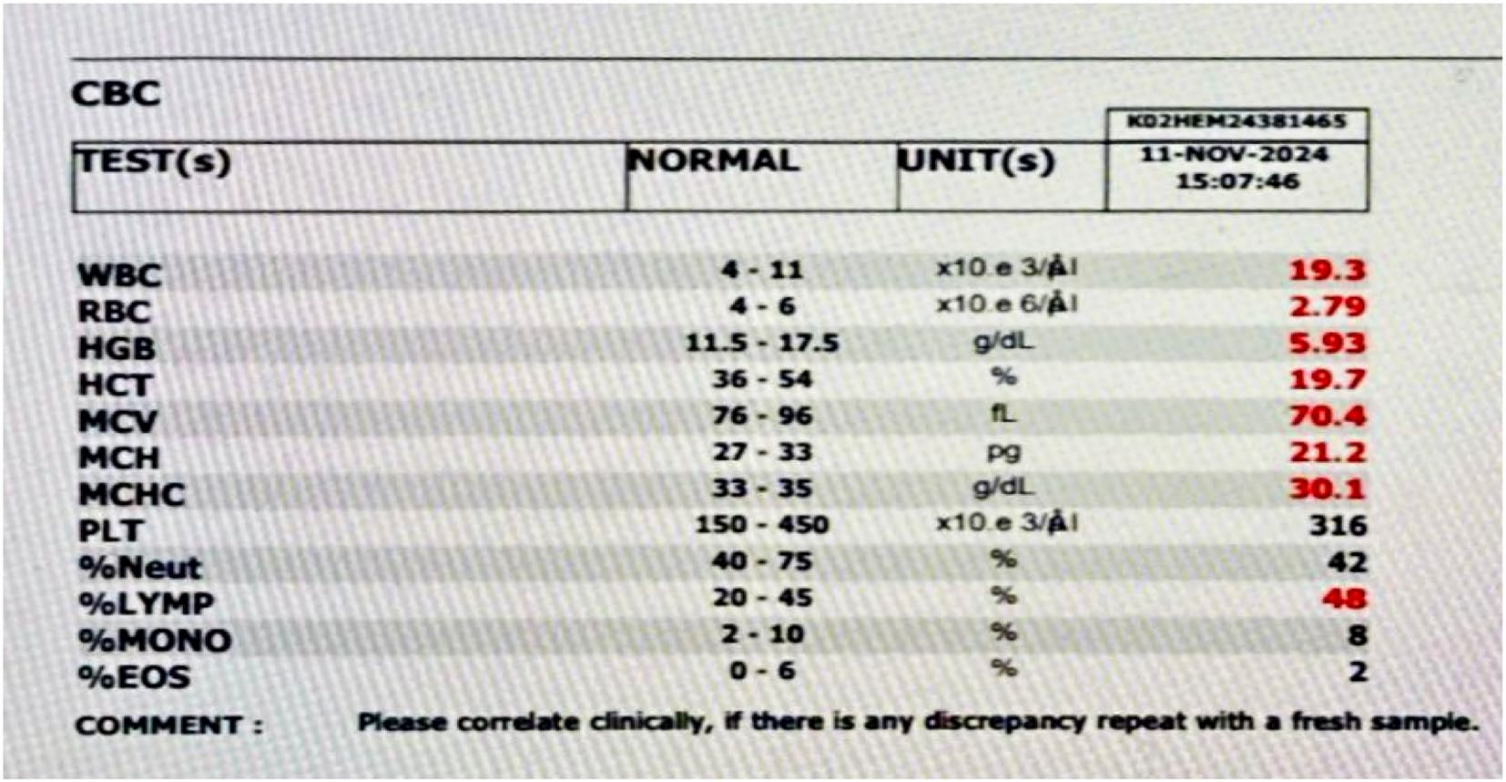
Complete blood count.

**FIGURE 2:**
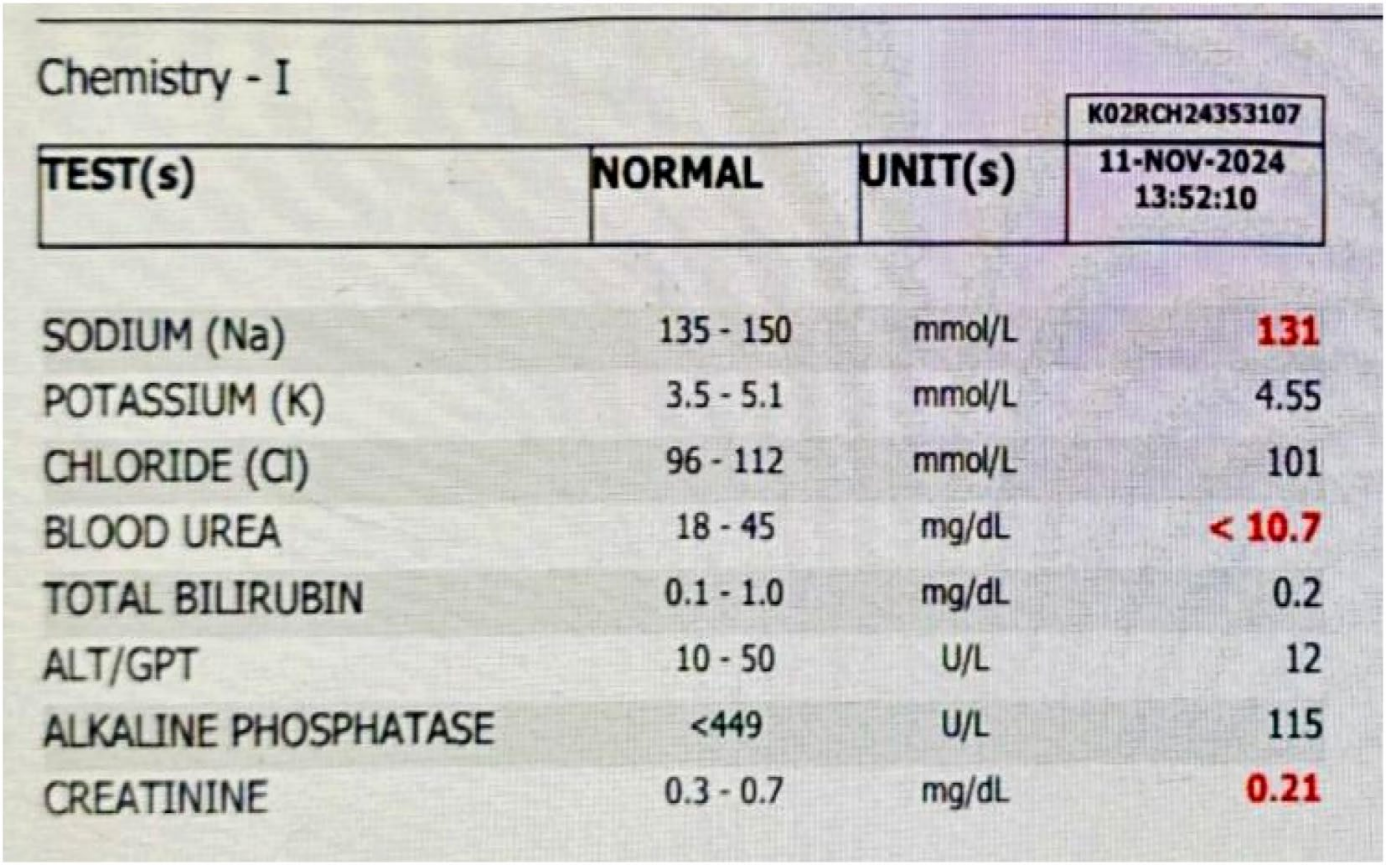
Serum electrolytes and liver function tests (LFTs).

**FIGURE 3:**
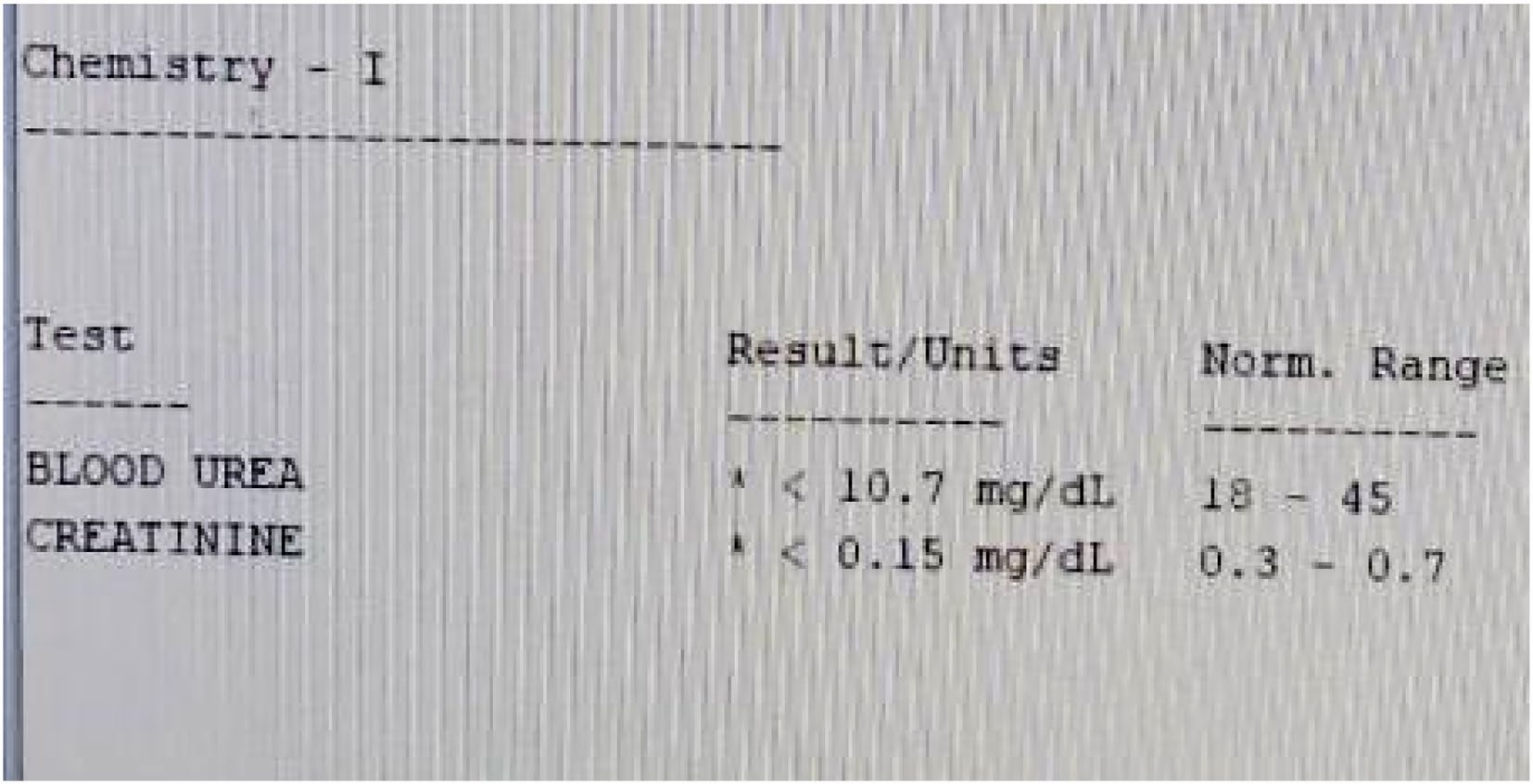
Renal function tests (RFTs).

Laboratory investigations, including a complete blood count (CBC), showed leukocytosis, predominantly lymphocytes, and microcytic anemia. Hemoglobin was 5.93 g/dL, as shown in (Figure 1). Liver function tests and serum electrolyte levels were performed; the only abnormality detected was hyponatremia, with a level of 131 mmol/L, shown in (Figure 2). In renal function tests, urea and creatinine levels were attributed to reduced muscle mass and dilutional effects in infancy and did not require specific intervention, as seen in (Figure 3). The child's constipation was managed with appropriate pediatric care, but the abdominal distension and mass raised suspicion of an underlying condition.

**FIGURE 4:**
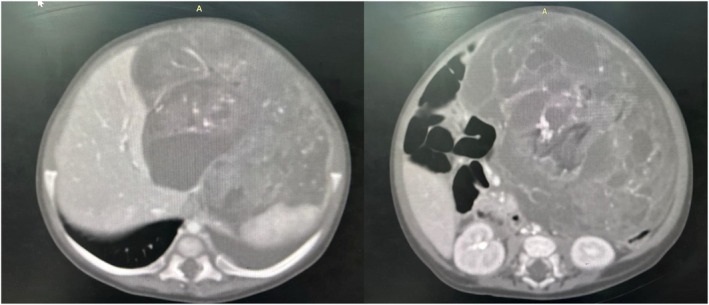
CT scan of the abdomen, showing a large, complex, heterogeneously enhancing lesion in the left hemiabdomen.

An abdominal ultrasound revealed a complex, heterogeneous mass in the upper abdomen. To better characterize the lesion, a contrast‐enhanced CT scan was performed, showing a large, complex mass originating from the greater curvature of the stomach. The mass was heterogeneous, with both solid and cystic components, consistent with a teratoma or another type of mixed tissue tumor. There was no evidence of metastasis or invasion into surrounding structures, as shown in (Figure 4).

**FIGURE 5:**
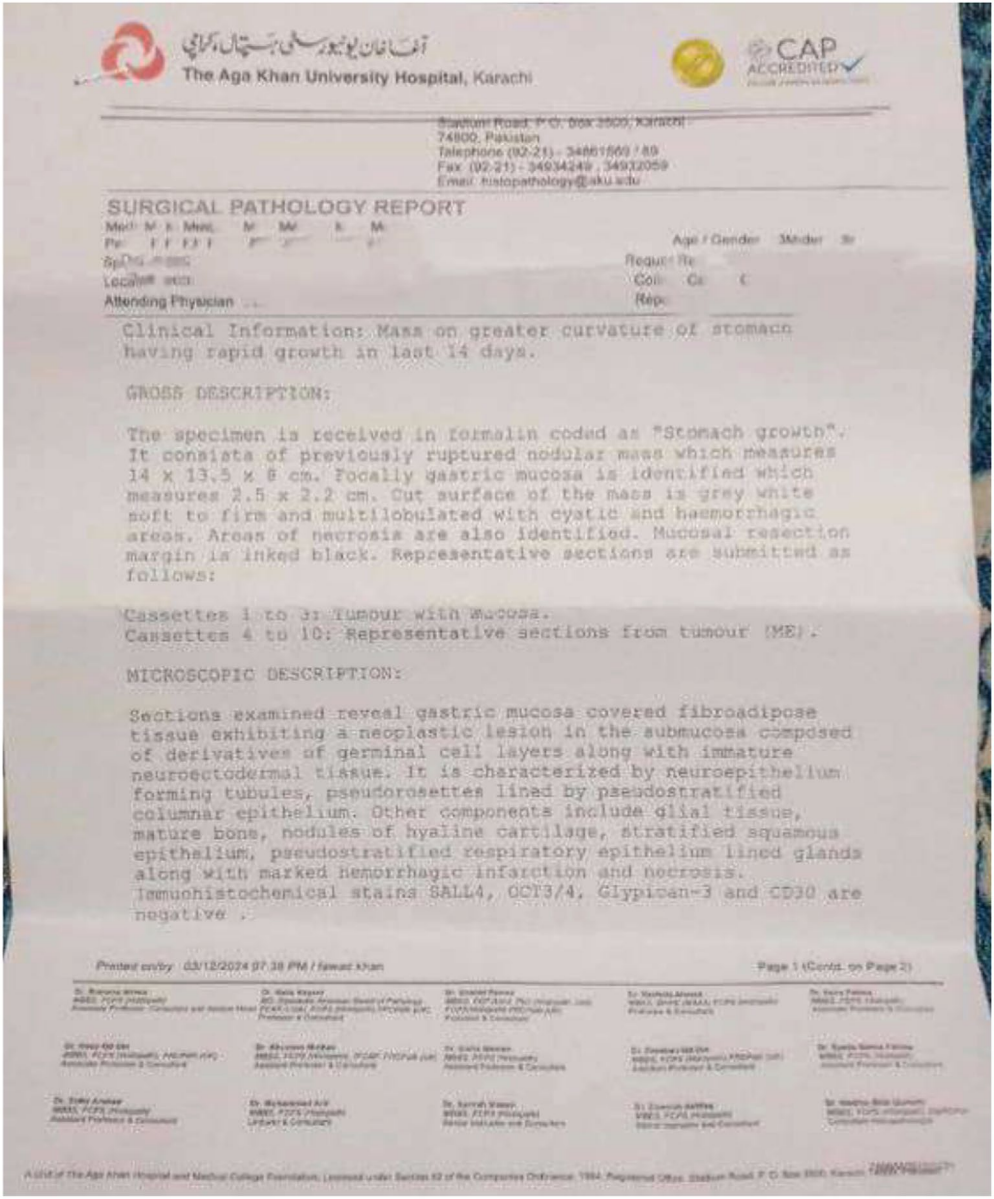
Report of histopathologic findings.

The resected mass was heterogeneous, comprising both solid and cystic components. The cystic areas contained sebaceous material, while the solid regions consisted predominantly of mature tissue elements. Microscopic examination demonstrated a teratomatous lesion composed of differentiated tissues derived from all three germ layers, including ectodermal components (keratinized epithelium with hair follicles), mesodermal elements (cartilage), and endodermal derivatives (gastrointestinal‐type epithelium). Focal immature neuroectodermal tissue was identified; however, no evidence of malignant transformation was observed. Based on the overall histomorphological features, the findings were consistent with a mature gastric teratoma (Figure 5). Histopathological photomicrographs were not available from the institutional records.

## 4 Treatment

**FIGURE 6:**
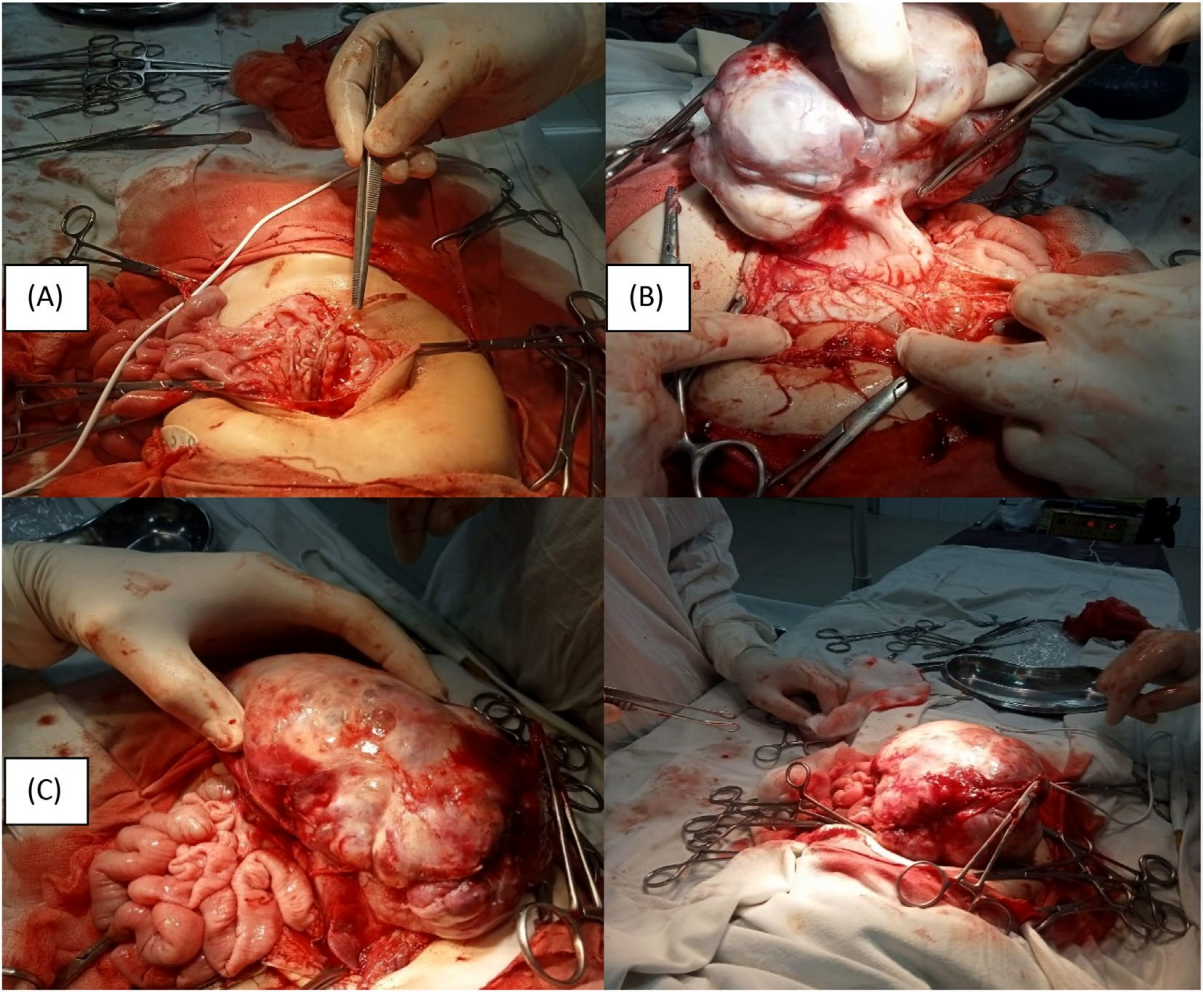
Surgical Intervention. (A) Performing the laparotomy. (B) Large, multilobulated, exophytic mass arising from the greater curvature of the stomach, with a smooth, vascular surface and variegated appearance suggesting areas of hemorrhage or necrosis. (C) The tumor was removed along with a small section of the adjacent gastric wall.

Given the size of the mass and the persistent symptoms, a decision was made to proceed with surgical exploration. A laparotomy was performed (Figure 6A), revealing a large, well‐defined mass originating from the greater curvature of the stomach. The tumor measured approximately 14 × 13 × 8 cm in size and was securely attached to the stomach wall (Figure 6B). The surrounding peritoneal surfaces appeared normal, with no signs of peritoneal seeding or distant metastasis. The tumor was removed along with a small section of the adjacent gastric wall through careful dissection to minimize damage to nearby tissues, as shown in (Figure 6C).

The infant's postoperative course was smooth. He started oral feeding on day 3 and symptoms improved, including a reduction in abdominal distension. The child was discharged on postoperative day 7 with a follow‐up scheduled in 6 weeks.

## 5 Conclusion

Gastric teratoma is an extremely uncommon tumor in infants, and its vague symptoms can delay diagnosis. This case underscores the importance of early imaging when an infant presents with progressive abdominal distension or unexplained upper abdominal fullness. Complete surgical excision provided definitive treatment in this case, and mature lesions generally carry an excellent prognosis, as evidenced by the patient's rapid recovery. Reported risks of recurrence or malignant transformation are derived from the literature, highlighting the importance of structured follow‐up.

## 6 Discussion

This case involves a young infant with increasing abdominal swelling and a noticeable gastric mass. It aligns with the typical presentation of gastric teratomas and underscores several vital points in diagnosis and management. Gastric teratomas are very rare in children and are usually detected during the neonatal period or early infancy. Their presentation, often accompanied by a growing abdominal mass and symptoms resulting from the mass's pressure, is well‐documented in the pediatric surgery literature [6]. These tumors originate from pluripotent germ cells and typically contain well‐formed elements from various germ layers, such as skin, hair, cartilage, and gastrointestinal mucosa. This composition explains the mixed solid and cystic appearance observed on imaging and the different tissue types identified during examination [7].

Clinically, early symptoms such as abdominal swelling, feeding difficulties, or vomiting are nonspecific, so a wide range of potential diagnoses must be considered. In infants, this may include enteric duplication cysts, neuroblastoma, hepatoblastoma, and other intra‐abdominal masses. However, specific imaging features, such as a mixed cystic and solid lesion with fat or calcifications, along with precise localization to the gastric wall, strongly suggest a teratoma and help narrow down the options [8]. Cross‐sectional imaging, like CT or MRI, is especially valuable because it reveals the organ of origin, helps identify fat or calcifications within the mass, and shows the relationship to surrounding organs. This information is essential for planning surgery aimed at preserving the stomach whenever possible [9].

Most mature gastric teratomas are not associated with elevated serum tumor markers. Elevated alpha‐fetoprotein (AFP) levels have been reported primarily in cases containing yolk‐sac or other malignant germ cell components. Although AFP assessment may be useful in selected cases pre‐ and post‐operatively, particularly when malignant elements are suspected, serum tumor markers were not measured in this patient, and postoperative follow‐up was guided by clinical assessment and imaging findings [10]. A definitive diagnosis is made through histopathology: identifying well‐formed derivatives from two or more germ layers confirms a mature teratoma. In contrast, immature elements or clear malignant transformation alter the prognosis and may require additional therapy.

Surgical removal with clear margins is the main treatment. Many gastric teratomas grow outward, allowing for organ‐sparing techniques such as local excision with part of the gastric wall and subsequent repair. However, larger or less ideally positioned tumors may require a more extensive approach. In this case, surgical management was tailored to the size and location of the tumor. While minimally invasive approaches have been reported in selected cases, open surgery was chosen to allow safe and complete excision. Complete tumor removal was achieved, which is considered important in reducing the risk of recurrence based on previously reported cases [11].

The prognosis after complete removal is very favorable for mature gastric teratomas, and many immature tumors also have positive outcomes when entirely excised. Ongoing monitoring is tailored based on the histology and preoperative marker levels. Routine follow‐up with periodic imaging during the early years after surgery is advisable, with the frequency and duration of monitoring depending on the initial histological grade and marker behavior [12].

## Author Contributions

**Syed Iftikhar Rahim:** conceptualization. **Mahnoor Nazir Afridi:** resources. **Arjun Singh:** resources. **Muhammad Waqas:** validation. **Sadaf Faryal:** visualization. **Ang Raj Karan:** supervision. **Asim Shah:** resources. **Suleman Khan:** writing – original draft. **Aizaz Anwar Khalid:** visualization. **Hammad Iftikhar:** methodology. **Mohammed Hammad Jaber Amin:** writing – review and editing.

## Funding

The authors have nothing to report.

## Disclosure

The CARE checklist was adhered to during our study.

## Ethics Statement

The authors have nothing to report.

## Consent

The patient provided informed consent to publish this case report, which includes relevant clinical details, diagnostic results, and treatment outcomes. The patient understands that all identifiable information will be kept confidential, and steps will be taken to protect their anonymity. They acknowledge that the report aims to improve medical knowledge and agree to its publication in a scientific journal. The patient was informed that participation is voluntary and that they can withdraw consent at any time before publication.

## Conflicts of Interest

The authors declare no conflicts of interest.

## Data Availability

The data used in this case report were obtained from a patient who presented to our hospital. All referenced studies and background information are publicly available on databases such as PubMed and Google Scholar. No additional datasets were generated or analyzed for this study.
